# Big data and artificial intelligence applied to blood and CSF fluid biomarkers in multiple sclerosis

**DOI:** 10.3389/fimmu.2024.1459502

**Published:** 2024-10-18

**Authors:** Georgina Arrambide, Manuel Comabella, Carmen Tur

**Affiliations:** Multiple Sclerosis Centre of Catalonia (Cemcat), Department of Neurology, Hospital Universitari Vall d’Hebron, Universitat Autònoma de Barcelona, Barcelona, Spain

**Keywords:** multiple scleorsis (MS), fluid biomarkers, demyelinating, machine learning and AI, deep learning

## Abstract

Artificial intelligence (AI) has meant a turning point in data analysis, allowing predictions of unseen outcomes with precedented levels of accuracy. In multiple sclerosis (MS), a chronic inflammatory-demyelinating condition of the central nervous system with a complex pathogenesis and potentially devastating consequences, AI-based models have shown promising preliminary results, especially when using neuroimaging data as model input or predictor variables. The application of AI-based methodologies to serum/blood and CSF biomarkers has been less explored, according to the literature, despite its great potential. In this review, we aimed to investigate and summarise the recent advances in AI methods applied to body fluid biomarkers in MS, highlighting the key features of the most representative studies, while illustrating their limitations and future directions.

## Introduction

Artificial intelligence (AI) techniques have proved very useful for the diagnosis and prognostication of several conditions around the world (1), including multiple sclerosis (MS) (2). AI methods used in medical research, including MS research, may include machine learning (ML) and deep learning (DL) analyses. Typically, while ML analyses are based on tabulated data as input to the model, DL models use raw data – typically images – as input to the model. Model outputs depend on the type of task that is needed, e.g., a given diagnosis (instead of another one), a certain disability milestone, or the presence of MRI activity in people who are receiving a given drug.

**Figure 1:**
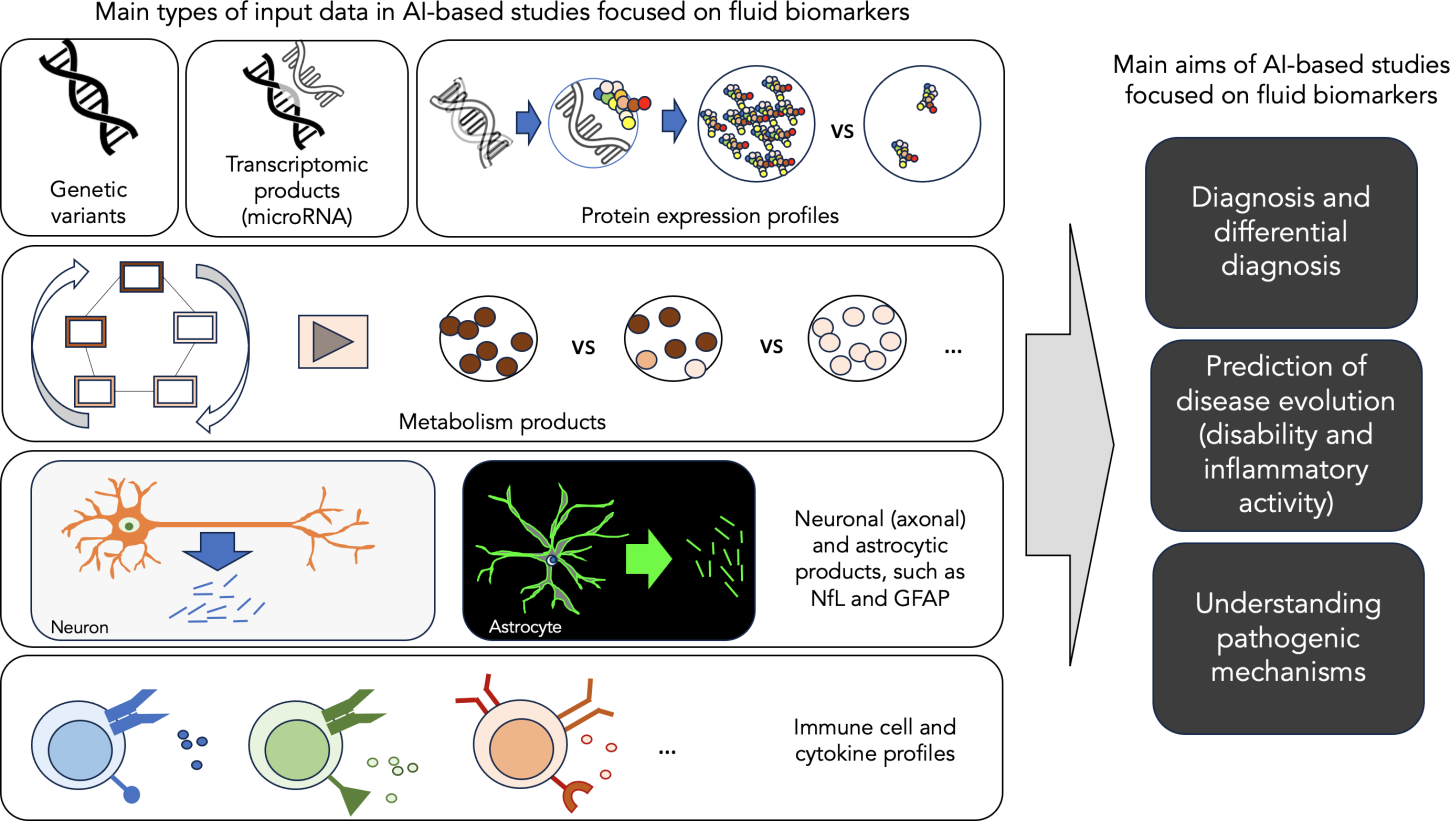
Main aims of AI-based studies focused on fluid biomarkers. This figure illustrates the main types of input data and the main aims of AI-based studies focused on fluid biomarker data in MS.

Multiple sclerosis (MS) is a chronic inflammatory-demyelinating condition of the central nervous system (CNS) with heterogeneous genetic and environmental risk factors (3). Disease diagnosis and monitoring strongly rely on routine clinical assessments and the use of conventional brain and spinal cord magnetic resonance imaging (MRI) as a biomarker. A biological marker, or biomarker, is a characteristic that is objectively measured and evaluated as an indicator of normal biological processes, pathogenic processes or pharmacologic responses to a therapeutic intervention (4). Besides MRI, body fluid biomarkers can also provide additional, independent data on MS. AI applications in MS can potentially help us better support the diagnosis, find markers for prognosis, facilitate accurate monitoring, and eventually understand the mechanisms of the disease. Focusing on these main challenges, this review aims to summarise the recent advances in AI applied to blood, serum and CSF biomarkers in MS, highlighting the key features of the most representative studies (**Figure 1**) (5). This review also aims to illustrate its limitations and future directions.

## Search strategy

**Figure 2:**
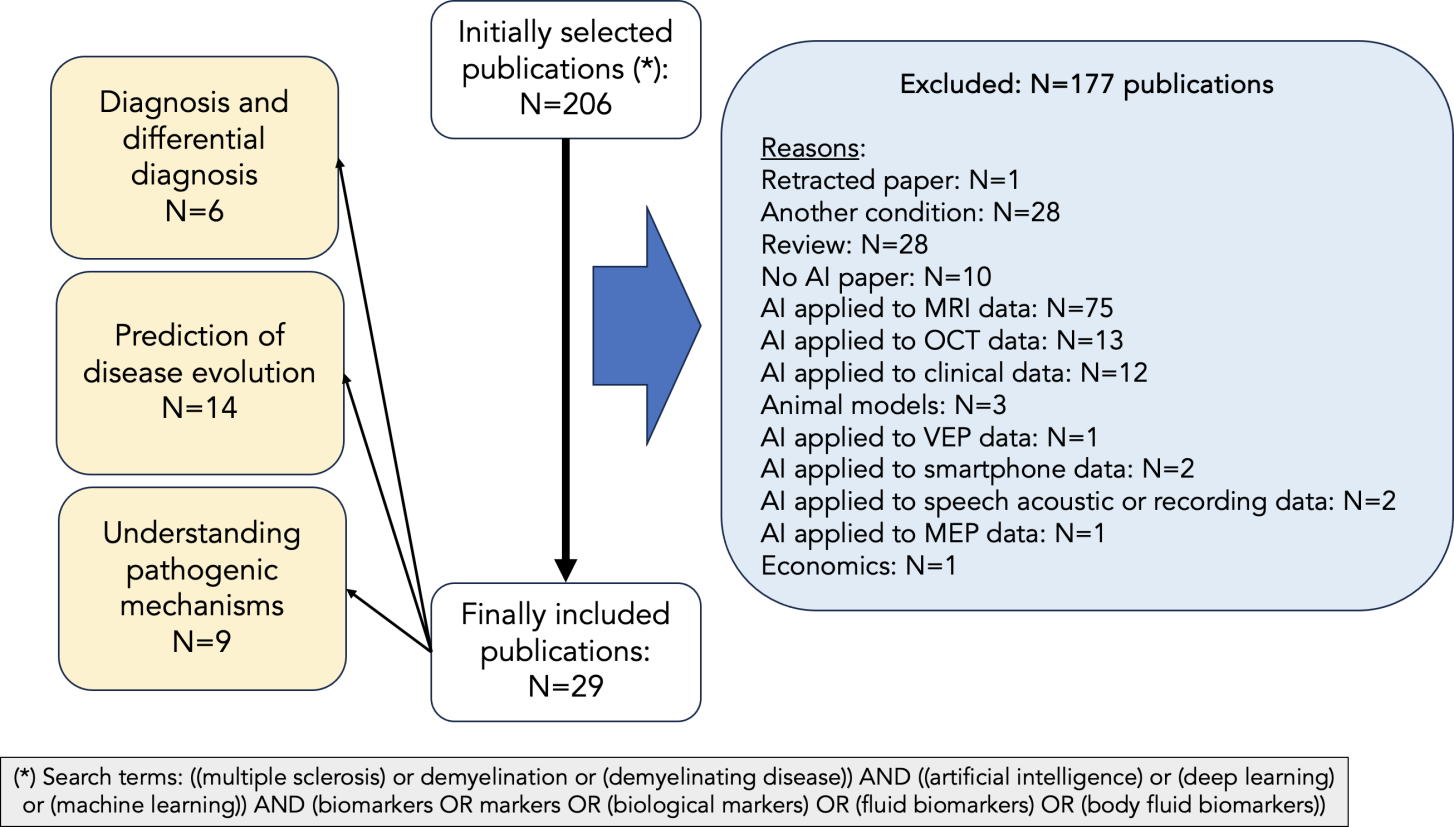
PRISMA chart describing article selection. We have followed a systematic approach for selecting the papers to be considered in our manuscript. After performing a PubMed search with the following terms: (multiple sclerosis or demyelination or demyelinating disease) AND (artificial intelligence or deep learning or machine learning) AND (biomarkers or markers or biological markers or fluid biomarkers or body fluid biomarkers), 206 records were obtained. Of those, only 29 were considered for this review after excluding those not meeting our inclusion criteria.

**Figure 3:**
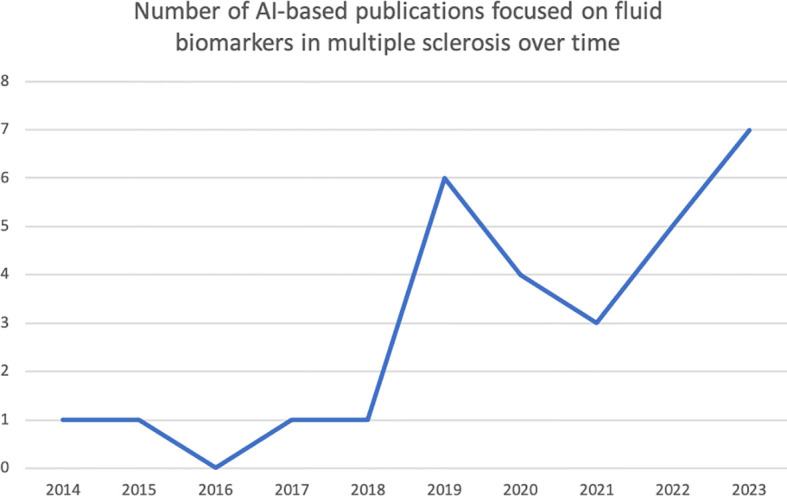
Distribution of the research papers on AI applied to biomarker data in MS over time. This histogram shows the number of research articles (of those 29 selected) published per year. It is to be noted that most of the papers have been published in the last 4 years.

We performed a search in PubMed based on the following criteria: (i) search terms: ((multiple sclerosis) or demyelination or (demyelinating disease)) AND ((artificial intelligence) or (deep learning) or (machine learning)) AND (biomarkers OR markers OR (biological markers) OR (fluid biomarkers) OR (body fluid biomarkers)); (ii) language of publication: English; (iv) type of paper: original research. For the purpose of this narrative review, we have focused on three aspects: (i) diagnosis & differential diagnosis; (ii) prediction of clinical outcome; (iii) understanding of pathogenic mechanisms. Thus, after the first literature search, we manually selected the papers if they were included in one of these three categories. Papers not clearly included in any of these categories were not considered in the review. Thus, we did not include papers whose main focus was methodological or animal research, and papers related to fluid biomarkers other than blood, serum and CSF. We also excluded review papers, editorials, and case reports. The PubMed search yielded 206 articles, published between 1996 (and especially between 2009) and 2024, both included (**Figure 2**). After excluding those not meeting our inclusion criteria, we revised 29 papers for their inclusion in this narrative review (**Figure 2**). Most of these papers have been published between 2019 and 2024 (**Figure 3**).

Once all papers were selected, they were divided into MS diagnosis and differential diagnosis (N=6), prediction of disease evolution (N=14), and understanding mechanisms of damage in MS (N=9). Of note, for some papers we found a degree of overlap and the decision to include them into one or another category depended on the main objectives described by the authors.

## MS diagnosis and differential diagnosis

The diagnosis of MS relies on integrating clinical, MRI, and laboratory findings and excluding alternative diagnoses, especially in the presence of red flags. Indeed, the diagnosis of MS is not devoid of challenges: other conditions may mimic MS, clinically or radiologically (6). In these circumstances, the use of AI algorithms may be useful (**Table 1**), especially in body fluid biomarker discovery studies such as those done with “omics” technology.

**Table 1:** Summary of selected studies focused on diagnosis and differential diagnosis.

Reference	Training and testing cohort, N	Independent validation cohort, N	Biomarker profiles	AI method: algorithms	Model input	Model output	Model performance	Comments
Pasella et al., Front Neuroinform. 2023 [ref (7)]	MS: n=299 (RRMS n=218, PPMS n=81) Healthy controls: n=619	0	Alleles responsible for HLA class I molecules and KIR genes, obtained from PBMC	DT	Genotyping for alleles at *HLA-A*, *-B*, *-C*, and *-DRB1* loci. Primers specific to 11KIR genes: *IR2DL1*, *KIR2DL2*, *KIR2DL3*, *KIR2DL5*, *KIR3DL1*, *KIR2DS2*, *KIR2DS3*, *KIR2DS4*, *KIR2DS5*, *KIR3DS1*	MS vs non-MS	identified 80.94% of MS patients in the training set and 73.24% in the validation set. Identified 71.08% of healthy controls in the training set and 66.07% in the validation set	Immunogenetic risk factors, specifically alleles responsible for HLA class I molecules and KIR genes, responsible for natural killer lymphocyte receptors
Guo et al., PLoS One. 2014 [ref (8)]	MS: n=26 OND: n=18	0	27336 probe sets obtained from gene expression profiles from the Array Express Database. Samples obtained from PBMC	SVM, ROC algorithm, Boruta algorithm	8 genes differentially expressed between MS and OND	MS vs OND	AUC 0.711-0.852. Accuracy of 86% in validation study	The 8 differentially expressed genes in MS vs OND were related to the protein kinase cascade, inactivation of MAPK, and regulation of signal transduction and apoptosis
Andersen et al., Mult Scler Relat Disord. 2019 [ref (10)]	Male subjects with MS: n=12 Male controls: n=13	0	Serum metabolites (lipid and amino acid profiles)	RF	12 metabolites	MS vs controls	6 metabolites with AUCs >80%: pyroglutamate, laurate, acylcarnitine C14:1, N-methylmaleimide, and 2 phosphatidylcholines (PC ae 40:5, PC ae 42:5)	Identified metabolites participate in glutathione metabolism, fatty acid metabolism and oxidation, cellular membrane composition, and transient receptor potential channel signalling. Their gene expression association suggested enrichment for pathways associated with apoptosis and mitochondrial dysfunction.
Lötsch et al., Sci Rep. 2018 (ref [11)]	MS: n=102 Healthy controls: n=301	0	43 lipid mediators from serum samples: ceramides (@)	Self-organising maps of neural networks, swarm intelligence and Minimum Curvilinear Embedding. In a second step, RF and computed ABC analysis-based feature selection	Classifier with 8 lipid biomarkers (GluCerC16, LPA20:4, HETE15S, LacCerC24:1, C16Sphinganine, biopterin, and endocannabinoids PEA and OEA)	MS vs healthy controls	98% accuracy for the 43 lipid mediators; classifier with ≥95% accuracy in training and test data sets	Most lipid mediator concentrations were reduced in MS. Exceptions were the ceramide LacCerC24:1 and the sphingolipid C16Sphinganin, found at higher concentrations in MS Cer16 and Cer24 might amplify cytokine-induced cell death of myelin-producing oligodendrocytes. HETE15S was shown to be regulated in CSF of MS patients. Enhanced activity of autotaxin was observed in serum samples of MS patients. PEA and OEA have been found in RRMS and SPMS. Neopterin is an activation marker of the innate immune system with increased levels in autoimmune diseases including the CSF of MS patients
Probert., et al. Front Immunol. 2021 [ref (12)]	MS with +OCB: n=41 Non-MS controls with +OCB: n=64 (*)	0	Metabolites and proteins in CSF	Multivariate OPLS-DA	8 metabolites significantly decreased in MS: 4 (myo-inositol, isoleucine, leucine, glutamine) had higher specificity than OCB for MS diagnosis. 9 biomarkers outperformed OCB as predictor of MS (CCN5, CDCC80, NTN1, vWF, DKK4, SOST, ERBB3, IGL4, and IGKV1-5). All significantly decreased in MS vs non-MS except for IGL4 and IGKV1-5, which were increased.	MS vs non-MS	The combination of CCN5, vWF, GFAP, and OCB status provided the best overall diagnostic properties (sensitivity 89%, specificity 92%, accuracy 91%) compared to OCB status	Integrative metabolomics and proteomic enrichment analysis revealed upregulated JAK-STAT and glycolysis pathways in MS, consistent with an increased inflammatory response and altered energy metabolism.
Gaetani et al., Int J Mol Sci. 2023 (ref [13)]	+OCB RRMS: n=58; -OCB RRMS: n=24; OND: n=36 (&)	0	Quantification of 92 immune activation CSF proteins	Hierarchical clustering to profile CSF proteins. Binomial and multinomial LASSO regressions to differentiate patient groups	92 tested proteins minus 45 with a call rate <85%, age, sex, NfL	MS vs OND; +OCB RRMS vs OND; -OCB RRMS vs OND	All: CD5 (AUC 0.87) and IL-12B (AUC 0.81). +OCB RRMS vs OND: IL-12B, CX3CL1, FGF-19, CST5, and MCP-1 (91% sensitivity, 94% specificity in the training set; 81% and 95%, respectively, in the validation set) -OCB RRMS vs OND: CX3CL1, CD5, CCL4, and OPG as well as NfL (87% sensitivity, 80% specificity in the training set; 56% and 48% in the validation set)	CD5 may act as a receptor in regulating T cell proliferation. IL-12B promotes differentiation of T cells into T helper 1 (Th1) cells. CX3CL1 increases IFN-γ and TNF-α gene expression and IFN-γ secretion by CD4+ T cells. FGF signalling may regulate inflammation and myelination in MS since an abundance. CST5 has shown potential as a relapse marker. MCP-1 may be involved in the recruitment of monocytes/macrophages and activated lymphocytes. CCL4 is involved in the disruption of the blood-brain barrier. OPG suppresses mRNA expression of CCL20, a chemokine involved in Th17 cell recruitment with anti-inflammatory effects
Martynova et al., Mediators Inflamm. 2020 [ref (14)]	MS: n=101 (RRMS n=49, SPMS n=31, PPMS n=21) and Non-MS subjects: serum n=101 and CSF n=25 ($)		45 leucocyte-activation regulatory cytokines measured in serum and CSF	k-Nearest Neighbour, DT, XGB, Gaussian Naïve Bayes and RF	22 cytokines altered in CSF and 20 in serum, 10 commonly affected in both (IL-1α IL-4, IL-18, CCL7, CCL27, CSF, IFN-γ, LIF, M-CSF, and TNF-α). Three independent datasets: cytokines affected both in CSF and serum, only in CSF and only in serum	MS vs non-MS	Diagnostic accuracy: ≥92% when any randomly selected 5 of any cytokines were used. The highest accuracy, 99%, obtained when including CCL27, IFN-γ, and IL-4	CCL27 could trigger T memory cells to produce IL-4 and IFN-γ. Interleukins and chemokines affected in serum and CSF could direct leukocyte migration targeting Th1 cells.

(*) Epilepsy (n=5), functional neurological disorder (n=12), gait disorder (n=1), meningitis (n=2), motor paresis (n=3), movement disorder (n=2), MG (n=2), neuralgic amytrophy (n=1), neuroinfection (n=3), normal pressure hydrocephalus (n=1), polyneuropathy (n=5), polyradiculitis (n=2), primary headache disorder (n=13), sensory disturbance (n=8), SLE (n=1), visual disturbance (n=1), white matter lesions/leukoencephalopathy (n=2); (&): headache: n=16; psychiatric disorders: n=13; mononeuropathy: n=4), dysmetabolic polyneuropathy (n=3); ($): tension type headache, residual encephalopathy, unspecified demyelinating disease of the CNS, cerebrovascular diseases, PML, migraine with aura; (@): Cer16:0, Cer18:0, Cer18:1, Cer20:0, Cer24:0, Cer24:1, GluCerC16:0, GluCerC24:1, LacCerC16:0, LacCerC24:0, LacCerC24:0); lyosophosphatidic acids (LPA16:0, LPA18:0, LPA18:1, LPA18:2, LPA18:3, LPA20:4); sphingolipids (sphinganine, sphingosine, S1P, SA1P C16Sphinganine, C18Sphinganine, C24Sphinganine, C24:1Sphinganine); prostaglandins (PGD2, PGF1α, PGE2, TXB2); dihydroxyeicosatrienoic acids (DHET5.6, DHET11.12, DHET14.15); hydroxyeicosatetraenoic acids (HETE 5 S, HETE_12S, HETE_15S, HETE_20S); endocannabinoids (AEA, OEA, PEA, 2-AG) and pterins (biopterin, neopterin); Abbreviations (in alphabetical order): AUC, area under the curve; CCL, chemokin (C-C motif) ligand; CCN5, connective tissue growth factor/cysteine-rich protein/nephroblastoma overexpressed-5; CD, cluster of differentiation; CDCC80, coiled-coil domain-containing protein 80; CSF, cerebrospinal fluid; CST5, cystatin D; CX3CL, chemokine (C-X3-C motif) ligand 1; DKK4, dickkopf-related protein 4; DT, decision trees; ERBB3, receptor tyrosine-protein kinase erbB-3; FGF, fibroblast growth factor; GFAP, glial fibrillary acidic protein; HLA, human leukocyte antigen; IFN, interferon; IGKV1-5, immunoglobulin kappa variable 1-5; IGL4, insulin growth factor-like family member 4; IL, interleukin; JAK-STAT, Janus kinase/signal transduction and transcription activation; KIR, killer immunoglobulin-like receptor; LASSO, least absolute shrinkage and selection operator regression; LIF, leukemia inhibitory factor; MAPK, mitogen-activated protein kinases; MCP, monocyte chemoattractant protein; M-CSF, macrophage colony-stimulating factor; MG, myasthenia gravis; MS, multiple sclerosis; NfL, neurofilament light chain; NTN1, netrin-1; OCB, oligoclonal bands; OND, other neurological diseases; OPG, osteoprotegerin; OPLS-DA, orthogonal partial least squares discriminant analysis; PBMC, peripheral blood mononuclear cells; PPMS, primary progressive multiple sclerosis; RRMS, relapsing remitting multiple sclerosis; ROC, receiver operating characteristic curve; RF, random forests; SLE, systemic lupus erythematosus; SOST, sclerostin; SPMS, secondary progressive multiple sclerosis; SVM, support vector machine; Th, T helper cells; TNF, tumor necrosis factor; vWF, von Willebrand factor; XGB, Extreme Gradient Boosting.

AI has been implemented to identify genetic susceptibility biomarkers. Pasella et al. (7) used decision trees (DT) to create a predictive tool assessing the likelihood of MS including alleles responsible for human leukocyte antigen (HLA) class I molecules and killer immunoglobulin-like receptor (KIR) genes, responsible for natural killer (NK) lymphocyte receptors. They studied 299 persons with MS (PwMS) and 619 healthy controls (HC). The algorithm accurately identified 80.94% of PwMS and 71.08% HC in the training set and 73.24% and 66.07%, respectively, in the validation set. Guo et al. (8) used Support Vector Machine (SVM) to identify gene expression profiles on the transcriptome of peripheral blood mononuclear cells (PBMC) from 26 PwMS and 18 subjects with other neurological diseases (OND). This approach identified 8 genes differentially expressed between groups with 86% accuracy in the validation study. These genes involved the protein kinase cascade, inactivation of mitogen-activated protein kinases (MAPK), and regulation of signal transduction and apoptosis.

The metabolomes of cells and tissues include lipids, amino acids, sugars and other molecules (9). Andersen et al. (10) used random forests (RF) to identify blood-based metabolite profiles that could discriminate between 12 male PwMS and 13 male controls. The top 6 candidate metabolites informative for MS, defined as having an area under the receiver operating characteristic (ROC) curve (ROC-AUC) >80%, participate in glutathione metabolism, fatty acid metabolism and oxidation, cellular membrane composition, and transient receptor potential channel signalling. Whilst metabolomics focuses on hydrophilic molecules, lipidomics has emerged as an independent “omics” due to its complexity (9). Lötsch et al. (11) used unsupervised ML to compare 43 lipid mediators in serum from 102 PwMS and 301 HC. The analyses showed 98% accuracy to differentiate PwMS from HC. Then, the authors used supervised ML implemented as RF and computed ABC analysis-based feature selection, to create a classifier. This approach identified 8 lipid biomarkers differentially expressed in PwMS with ≥95% accuracy in training and test datasets.

Other studies have focused on CSF biomarkers. Probert et al. (12) used ML to profile metabolites and proteins in CSF samples from 41 PwMS and positive IgG oligoclonal bands (+OCB) and 64 patients with OND and +OCB. Multivariate orthogonal partial least squares discriminant analyses (OPLS-DA) showed that combining connective tissue growth factor/Cysteine-rich protein/Nephroblastoma overexpressed-5 (CCN5), von Willebrand Factor (vWF), glial fibrillary acidic protein (GFAP), and OCB provided the best diagnostic properties to discriminate MS from OND (89% sensitivity, 92% specificity, 91% accuracy). Gaetani et al. (13) used hierarchical clustering to profile 92 immune activation CSF proteins in +OCB relapsing-remitting MS (RRMS) (n=58), -OCB RRMS (n=24), and OND (n=36). Next, they used binomial and multinomial least absolute shrinkage and selection operator (LASSO) regressions to differentiate among these groups. Cluster of differentiation 5 (CD5) (ROC-AUC 0.87) and interleukin 12B (IL-12B) (ROC-AUC 0.81) were the best MS vs OND predictors. The model that best differentiated +OCB RRMS from OND included IL-12B and 4 other proteins (sensitivity 91% and 81%, specificity 94% and 95% in the training and validation sets, respectively). The model that best differentiated -OCB RRMS from OND included CD5, 3 other immune activation proteins as well as NfL, assessed additionally (sensitivity 87% and 56%, specificity 80% and 48% in the training and validation sets, respectively).

One study assessed proteins in both CSF and serum. Martynova et al. (14) used five ML models to study differences in 45 leucocyte-activation regulatory cytokines, measured in serum and CSF of 101 PwMS and in 101 serum and 25 CSF samples from non-MS subjects. Twenty-two cytokines were altered in CSF and 20 in serum, of which 10 were commonly affected. Next, three independent datasets including cytokines affected in CSF and serum, only in CSF, and only in serum were used as input to ML models to predict MS. Diagnostic accuracy was ≥92% when any randomly selected five of any cytokines were used.

## Prediction of MS evolution

The high heterogeneity of MS in terms of disease evolution means that the prognostication in clinical practice is extremely difficult. Although the presence of a high number of inflammatory-demyelinating lesions in the brain (15), and the presence of infratentorial (16), cortical (17), spinal cord (18), lesions at the time of the first attack are well-known predictors of a worse clinical evolution, these associations are only meaningful at a group level. That is, the prediction of the disease at the individual level based on these known predictors is still far from optimal. For that reason, over the years, a number of authors have aimed at predicting MS evolution based on these factors but through the development of AI models, with a much greater potential – at least theoretically – than classical statistical models. In spite of this, though, the ability to currently build (and publish) AI models to predict disease evolution based on MRI and clinical data is still limited. This limited ability becomes evident especially when a model built in a given cohort is applied in a completely unseen, independent, validation cohort, showing a much lower accuracy than expected (much lower than that of the original cohort). This possibly suggests that the variability across people with MS is probably larger than what we thought and that mismatches between accuracies in original (training and testing) cohorts and external validation cohorts may be due to an overfitting of the data by the model in the original cohorts. Additionally, this may also suggest that other aspects apart from MRI and clinical data may be playing a role in the evolution of the disease. Over the last 10 but especially over the last 5 years, some studies using AI models applied to biomarker data to explain concurrent and future disease evolution have started to emerge (**Table 2**).

**Table 2:** Summary of selected studies focused on prediction of disease course: relapses and disability accumulation.

Reference	Training and testing cohort, N	Independent validation cohort, N	Follow-up time (study design)	Biomarker profiles	AI method: algorithms	Model input	Model output	Model performance	Comments
Cross-sectional prediction*									
Flauzino et al., Metab Brain Dis. 2019 [ref (19)]	122 patients with MS, i.e., RRMS, N=103; PPMS, N=3; SPMS, N=16	0	NA	Serum biomarkers including immune-inflammation, metabolic, and nitro-oxidative stress features	Multilayer perceptron neural network	Immune inflammatory (Th17/Treg ratio), metabolic (LDL/HDL ratio, uric acid, homocysteine) and oxidative stress (lipid hydro-peroxides, carbonyl protein, AOPP, NO metabolites) biomarkers, together with age, sex, disease duration, body mass index, and presence of metabolic syndrome	Disability status based on EDSS score: i) ≥3.0 vs <3.0 (binary outcome) ii) as a continuous outcome	ROC AUC = 0.842	Immune inflammatory, metabolic and oxidative stress pathways play a key role in disability accumulation in MS
Jackson et al., Ann Hum Genet. 2020 [ref (22)]	205	94	NA	113 genetic variants previously identified as related to MS severity	Random forest regression	19 genetic variants (GeM-MSS model)	MS-DSS, a score defined through a statistical model which takes into account CNS damage and demographic features [ref (46)]	GeM-MSS RMSE (error) = 0.464	The 19 genetic variants included in the GeM-MSS are related to 12 genes associated with immune cell regulation, complement activation and functions of neurons
Brummer et al., Brain Commun. 2022 [ref (21)]	152 patients with early MS	101 early MS	NA	Serum NfL	Support vector regression	Serum NfL, lesion volume, grey matter volume	Cognitive status based on SDMT score (continuous outcome)	Accuracy = 90.8%, greater than the accuracy of the models with individual predictors	The combination of blood and imaging measures improves the accuracy of predicting cognitive impairment
Zhu et al., Brain Commun. 2023 [ref (30)]	431	0	NA	19 serum protein biomarkers: APLP1, CCL20, CD6, CDCP1, CNTN2, CXCL9, CXCL13, FLRT2, GFAP, MOG, NfL, OPG, OPN, PRTG, SERPINA9, TNFSF10A, TNFSF13B, VCAN	LASSO, Random forest, Extreme Gradient Boosting, Support Vector Machines, stacking ensemble learning	7 clinical factors (age at sample collection, sex, race/ethnicity, disease subtype, disease duration, DMT, and time interval between sample collection and closest PRO assessment) and 19 serum protein biomarkers	Disability status based on PDDS score: ≥4 vs <4 (binary outcome) PDDS score: as categorical variable	ROC AUC = up to 0.91 (for LASSO prediction of PDDS using combined clinical and biomarker profiles as input)	Combined (clinical + biomarkers) models: the best LASSO better than other ML approaches Serum multi-protein biomarker profiles: better than single-protein (e.g., NfL or GFAP) models
Longitudinal prediction (**)									
Ebrahimkhani et al., Mol Neurobiol. 2020 [ref (32)]	29 RRMS patients who were about to start on fingolimod	0	0.5 years (6 months); however, the study does not focus on future but concurrent prediction (i.e., disease activity and microRNA dysregulation occur over the same period of time)	Exosome miRNAs	Random forest	Out of all micro-RNAs, 15 were selected for being dysregulated between active and non-active patients, 6 months after fingolimod onset. Of those, 11 were selected for having ROC AUC 95%CI above 0.50. Then, out of a total of 2037 combinations of these 11 microRNAs, 3 combinations ($) were chosen for their highest accuracy	Disease activity vs no activity, based on MRI, i.e., presence of gadolinium-enhancing lesions (binary outcome)	Prediction accuracy (of combined microRNAs) = 0.92	microRNA signatures are noninvasive biomarkers which may help predict treatment response in the future
Baranzini et al., Mult Scler. 2015 [ref (27)]	155 RRMS on beta-interferon treatment	0	0,77 years (40 weeks)	Gene expression profiles at treatment onset or over the follow-up (i.e., *induction ratios* of gene expressions after treatment onset)	Random forest	Triplet (3-gene) expression profiles (several triplet combinations were assessed)	Disease activity free on treatment (presence of clinical and/or MRI activity) vs suboptimal response (binary outcome)	Predictive accuracy = 0.59-0.68 ROC AUC = up to 0.63	Future (IFNb) treatment response may be predicted with gene expression profiles at treatment onset or over the first weeks after that, using models of machine learning
Waddington et al., Front Immunol. 2020 [ref (33)]	89 patients with RRMS/first demyelinating attack who were about to start on beta-interferon treatment	0	1 year	156 serum metabolites (see paper for full details)	Random forest, support vector machine, and LASSO logistic regression (K-nearest neighbour and decision trees also tested for comparison)	60 and 59 serum metabolites (out of 156) at baseline (before IFNb onset) and after 3 months, respectively; the remaining 96 and 97 metabolites, respectively, were excluded because of a strong correlation between them and the finally chosen 60 and 59 ones	ADA positive, i.e., i) bAbs+ & nAbs+ or ii) bAbs- but nAbs+ and titer ≥ 320 U/mL, within 12 months of starting treatment, vs ADA negative (binary outcome)	Classification accuracy (baseline) = 0.695-0.854 Classification accuracy (3 months after IFNb onset) = 0.712-0.863	ADA status may be predicted through serum metabolites
Herman et al., iScience. 2023 [ref (29)]	123	56	1 year	498 CSF metabolites	Elastic-net regularized classifier model In addition, *conformal prediction* analyses provides confidence in individual patient predictions	CSF metabolites: out of 498, 15 metabolites are selected	MS phenotype: PMS vs RRMS (binary outcome)	ROC AUC = 0.93, better than any of the single metabolic features in isolation	This study provides confidence in individual patient prediction (=0.88), which can help with patient monitoring
Andorra et al., J Neurol. 2023 [ref (23)]	322	271	2 years	Genomics: MS-associated (HLA and non-HLA) SNPs; Cytomics: levels of effector and regulatory T cells, B cells, and NK cells; Phospho-proteomics: 25 kinases participating in pathways associated with MS	Random forest	Brain MRI, OCT, and multiomics (genotyping, cytomics and phospho-proteomics) from PBMC	CDA on different scales (EDSS, T25WT, 9HPT, SDMT, SL25, HCVA) vs no-CDA (binary outcomes); NEDA vs no-NEDA (binary outcome); MSSS, ARMSS, onset of DMT, escalation from low- to high-efficacy DMT (continuous outcomes)	ROC AUC = from 0.50 (T25WT-CDA) to 0.81 (SL25-CDA); Balanced accuracies = from 0.5 (9HPT or T25WT) to 0.69 (starting therapy) Sensitivities = almost all between 0.82 and 0.94 PPVs = almost all between 0.8 and 0.9	Models provided better sensitivities and PPVs than accuracies or AUC; Models including imaging & genetics or omics slightly improved model performance (with respect to models with clinical predictors only) and only in 50% of the times
Ferrè et al., J Pers Med. 2023 [ref (24)]	304 patients on fingolimod treatment	77 patients on fingolimod treatment	2 years	Genetic data	Random forest	123 SNPs (*genetic model*), clinical data (*clinical model*), or both (*combined model*)	NEDA vs no-NEDA (binary outcome)	ROC AUC genetic model = 0.65 ROC AUC combined (genetic and clinical) model = 0.71	ML models integrating clinical and genetic data can help predict disease evolution in pwMS on fingolimod
Fagone et al., Mol Med Rep. 2019 [ref (26)]	12 patients with RRMS who were about to start on natalizumab	0	3 years	Whole−genome expression data from CD 4+ T cells (assessed before natalizumab onset)	UnCorrelated Shrunken Centroid Algorithm (¢)	Genetic expression of 17 genes related to CD4+ T cells	Disease activity or not, based on presence (vs absence) of relapses over the whole follow-up of 3 years (binary outcome)	Accuracy = 0.892	Gene expression profiles may help design personalised therapeutic strategies for patients with MS
Uphaus et al., EBioMedicine 2021 [ref (28)]	196 patients with RRMS/ first demyelinating attack	204 RRMS/ first demyelinating attack	Median: 6 (IQR 4.3-7.5) years	Serum NfL	Support vector machine	Serum NfL levels at baseline and ratio NfL follow-up/baseline +/- age & T2 lesion number at baseline	Relapse-free progression (binary outcome); Transition to SPMS (binary outcome)	For relapse-free progression: ROC AUC = 0.811 (NfL + age & T2 lesion number) For SPMS transition: ROC AUC = 0.651	Serum NfL levels may help predict future relapse-free progression in clinical practice, together with age and T2 lesions at baseline
Everest et al., PLoS One. 2023 [ref (31)]	94	40	Mean: 8.2 ± 2.2 years	CSF proteomics data: 151 differentially expressed CSF proteins, including C3bCfb, A2M, ATF7, PRBP, Haptoglobin, PDS5B, Myosin, CD36, and ApoA1 (ref (47)]	Genetic algorithm (Holland J. Adaptation in natural and artificial systems. University of Michigan Press, 1975)	CSF proteomics data	Disease severity status (binary outcome) based on ARMSS score on last follow-up: ≥5 (unfavourable group) vs <5 (favourable)	Rule 1 (to select ARMSS≥5): ROC AUC = 86.34% Rule 2 (to select ARMSS<5): ROC AUC = 73.26%	Novel candidate CSF protein biomarkers are proposed, to be validated in larger samples
Campagna et al., Clin Epigenetics. 2022 [ref (25)]	235 female patients with RMS	0	Median: 11.13 (IQR 9.49; 12.59) years	DNA methylation data assessed through Illumina methylation EPIC array	Elastic-net regression and logistic regression	Clinical data (age and symptoms), DNA methylation data of genes related to neuronal structure and function	Disease severity status (binary outcome) based on ARMSS score: mild vs severe (i.e., median ARMSS score below or above 20^th^ or 80^th^ percentile, respectively, of the cohort)	Methylation model ROC AUC = 0.91 (vs clinical model ROC AUC = 0.74)	Whole-blood methylation can predict disease severity in RMS and seems to affect genes related to neuronal structure and function

(*) Articles shown in chronological order; (**) Articles shown based on length of follow-up; (¢) UC SC; http://home.cc.umanitoba.ca/~psgendb/birchhomedir/BIRC HDE V/doc/MeV/manual/usc.html; ($) Combination 1: miR-432-5p and miR-485-5p; combination 2: miR-432-5p, -485-5p, -375; combination 3: miR-432-5p, −485-5p, −134-5p; Abbreviations (in alphabetical order): 9HPT, 9-hole peg test; A2M, alpha-2-macroglobulin; ADA, anti-drug antibodies; AOPP, Advanced oxidation protein products; APLP1, amyloid beta precursor like protein 1; ApoA1, apolipoprotein A1; ARMSS, age-related MS severity scale; ATF7, cyclic AMP-dependent transcription factor ATF-7; AUC, area under the ROC curve; bAbs, IFNb-binding antibodies; C3bCfb, chain F, crystal structure of complement C3b in complex with factor B; CCL20, chemokine (C-C motif) ligand 20; CD6, cluster of differentiation 6; CDA, confirmed disability accumulation; CDCP1, CUB-domain-containing protein 1; CNTN2, contactin-2; CXCL13, chemokine (C-X-C motif) ligand 13; CXCL9, chemokine (C-X-C motif) ligand 9; DMT, disease modifying treatment; EDSS, Expanded Disability Status Scale; FLRT2, fibronectin leucine-rich transmembrane protein 2; GFAP, glial fibrillary acidic protein; HCVA, high contrast vision; IFNb, interferon beta; IL12B, interleukin-12 subunit beta; IQR, interquartile range; LASSO, Least Absolute Shrinkage and Selection Operator; miRNA, microRNA, which are small, non-coding RNA molecules; MOG, myelin oligodendrocyte glycoprotein; MS, multiple sclerosis; MS-DSS, MS disease severity scale, defined thanks to a statistical model [ref (46)] which takes into account, the amount of CNS-tissue destruction measured by Combinatorial MRI scale of CNS tissue destruction (COMRIS-CTD) [ref (43)], and demographic data; MSSS, multiple sclerosis severity scale; Myosin, human skeletal mRNA for myosin heavy chain light meromyosin region; N0, sample size of the training and testing cohort; N1, sample size of the validation cohort; NA, not applicable; nAbs, IFNb-neutralising antibodies; NEDA, no evidence of disease activity; NfL, neurofilament light chain; OPG, osteoprotegerin; OPN, osteopontin; PBMC, peripheral blood mononuclear cells; PDDS, patient-determined disease steps; PDS5B, human androgen-induced prostate proliferative shutoff associated protein (AS3); PMS, progressive MS; PPV, positive predictive value; PRBP, plasma retinol binding protein; PRO, patient-reported outcome; PRTG, protogenin; RRMS, relapsing-remitting MS; SDMT, Symbol Digit Modality Test; SERPINA9, serpin family A member 9; SL25, 2.5% low contrast visual acuity; SNPs, single nucleotide polymorphisms; T25WT, timed 25 feet walking test; TNFSF10A, tumor necrosis factor ligand superfamily member 10; TNFSF13B, tumor necrosis factor ligand superfamily member 13B; VCAN, versican.

Regarding the studies that have focused on the concurrent prediction of clinical outcomes, in 2019, Flauzino et al. (19), published a study where 122 people with MS were tested on several serum biomarkers to predict concurrent disability status. These biomarkers, which were related to the immune-inflammatory response, lipid and protein metabolic pathways, and oxidative stress, were able to predict which patients had an Expanded Disability Status Scale (EDSS) (20) score above or below 3.0 with high accuracy (Area under the ROC curve = 0.842). These results suggest that Immune inflammatory, metabolic and oxidative stress pathways may play a key role in disability accumulation in MS and deserve further research. In another interesting study focused on concurrent prediction, Brummer and colleagues (21) showed how serum neurofilament light (NfL) levels could improve our ability to detect cognitive dysfunction, especially when added to MRI predictors such as grey matter volume. The authors of this study not only built a ML model with high predictive accuracy, but also validated the ML model in an external cohort, supporting the generalisability of the model (21). Finally, we highlight the paper from Jackson and colleagues (22), where ML models based on random forest regression were built to predict a multi-dimensional score of disease severity using genetic variants previously identified as related to MS severity. Interestingly, the results, which could be validated in an external cohort, showed that the 19 most predictive genetic variants were located in 12 genes associated with immune cell regulation, complement activation and functions of neurons (22). This supports the robustness of the results while providing important insights on the mechanisms of progression in MS.

Regarding the studies with a longitudinal design, there is a high variability in terms of the length of the prediction period, ranging from 6 months to 11 years, and in terms of the nature of the predictor data, i.e., the input of the ML model. For instance, there are studies which have used genetic data, focusing on the presence of certain genetic variants or single nucleotide polymorphisms (SNPs) (23, 24). Other studies have focused instead on the presence of certain epigenetic mechanisms, such as DNA methylation (25), and on certain gene expression profiles (26, 27). Also, a few studies have demonstrated the ability of (immune) cellular profiles to predict clinical outcome (23). Finally, there are studies which have based their predictions on the presence of specific serum and CSF proteins and metabolites (28, 29). In relation to the output data, i.e., the outcome of the ML model, most studies focus on disability progression measures (19, 21–23, 25, 28, 30, 31), although some of them have chosen acute activity (generally MRI activity) outcomes (24, 26, 27, 32) and one focused on the development of anti-drug neutralising antibodies (33), known to reduce the effectiveness of the disease-modifying drug (33).

In relation to the studies which have used SNP data to predict future outcome, the article by Andorra et al. (23) is of special interest. In this study, not only SNPs located in Human Leukocyte Antigen (HLA) and non-HLA genes were considered as predictors, but also data on immune cell populations, proteomics, brain MRI, and optic coherence tomography (OCT) data. In this study, whose results were validated in an external cohort, the authors predicted the development of confirmed disability accumulation on different disability outcomes after 2 years of follow-up, with high sensitivity (23).

Among the studies with longest predictive periods, there is the paper by Uphaus et al. (28), which used NfL data to predict 6-year development of relapse-free progression and transition from RRMS to SPMS with high accuracies, especially for the former outcome and especially when combined with age and T2 lesion volume (28). More recently, Everest et al. (31) published a paper where CSF proteomics data was used to predict unfavourable evolutions over an 8-year follow-up period (on average) with very high accuracies. In this paper, which included an external validation analysis, the authors propose several novel candidate CSF protein biomarkers with a promising future in disease prediction modelling (31). Finally, Campagna et al. (25) exploited the DNA methylation profiles of 235 women with MS to predict disease severity over an 11-year period, again with high accuracy. Although this model was not externally validated in an independent cohort, the length of its prediction and the nature of the biomarker used make it especially relevant. Interestingly, those genes with greater levels of methylation seemed to be related to neuronal structure and function (25).

## Investigation of disease mechanisms

The pathophysiological processes in MS are not completely understood and are believed to be highly heterogeneous across people and disease stages. Fluid biomarker studies using AI to understand pathogenetic mechanisms could contribute to a greater characterisation of MS by expanding the concept of classical phenotypes (**Table 3**).

**Table 3:** Summary of selected studies focused on disease mechanisms.

Reference	Training and testing cohort, N	Independent validation cohort, N	Biomarker profiles	AI method: algorithms	Model input	Model output	Model performance	Comments
Acquaviva et al., Cell Rep Med. 2020 [ref (34)]	313 subjects: CIS (n=57), RRMS (n=108), SPMS (n=26), PPMS (n=35), OND (n=27) Healthy subjects (n=60)	0	Transcriptomic profiles of PBMCs	Training set: nested cross-validation Validation set: ward DT-based algorithms (RF, FTs and ADAboost-FT)	Raw and processed microarray data from the GEO database, age, sex	MS classifiers: MS vs non-MS Relapsing vs progressive MS	MS vs non-MS: on 139 probes, 94.3% sensitivity and 87.5% precision. Relapsing vs progressive MS: 222 probes, 83.3% sensitivity and 93.8% precision. PPMS vs RRMS: 266 probes, 90% sensitivity and 90% precision. SPMS vs RRMS: 201 probes, 87.5% sensitivity and 100% precision	Identified transcripts in MS vs non-MS: related to interferon signalling, chromatin remodelling and apoptosis. Identified transcripts in relapsing vs progressive MS: related to cell cycle and T cell activation for both progressive forms; protein ubiquitination, cell migration, and fatty acid metabolism for PPMS; and regulation of GTPase activity, locomotor behaviour, and blood coagulation in the SPMS signature.
Sun et al., Front Genet. 2022 [ref (36)]	miRNA-MS associations from the disease-related miRNA from the HMDD. MS-related miRNAs as positive samples, and randomly selected associations with n times the number of positive samples from unlabelled miRNAs associations as negative samples, where n∈(2,10,20,30,40,50)	0	MS-related miRNAs	CNN vs DT, SVM, logistic regression, and GaussianNB	miRNAs	Top 10 predicted miRNAs: hsa-miR-605-5p, hsa-miR-15b-5p, hsa-miR-16-5p, hsa-miR-17-5p, hsa-miR- 181a-5p, hsa-miR-181b-5p, hsa-miR-181c-5p, hsa-miR-18a-3p, hsa-miR-195-5p, and hsa-miR-196a-5p.	ROC-AUC 0.87 with CNN	Some of the miRNAs were differentially expressed in RRMS or related to Th17 cell differentiation; one of them (miR-16-5p) decreased in PBMCs after initiation of therapy with interferon β
Lötsch et al., Int J Mol Sci. 2017 [ref (38)]	MS: n=102 Healthy subjects: n=301	0	3 types of lipid biomarkers in serum: eicosanoids: n=11; ceramides: n=10; and lysophosphatidic acids: n=6	ESOM combined with the U*-matrix visualisation technique	Eicosanoids, ceramides and lysophosphatidic acids	Data structures in eicosanoid and ceramide serum concentrations	Ecosanoid concentrations: sensitivity 54%, specificity 100%, accuracy 77%. Ceramid concentrations: sensitivity 89.2%, specificity 100%, accuracy 94.6%.	Lipid metabolism has been suggested to play a critical role in the pathophysiology of MS, influencing inflammation, neurodegeneration, myelin damage, and repair processes
Mezzaroba et al., Mol Neurobiol. 2020 [ref (39)]	MS: n=174 (CIS n=5; RRMS n=144, SPMS n=20, PPMS n=5) Controls: n=182	0	Plasma levels of TNF-α, sTNFR1, sTNFR2, adiponectin, hydroperoxides, AOPP, nitric oxide metabolites, TRAP, SH groups, and serum levels of zinc	NNA and RBF/SVM	TNF-α, sTNFR1, sTNFR2, adiponectin, hydroperoxides, AOPP, nitric oxide metabolites, TRAP, SH groups, and zinc	MS vs controls	Low concentrations of four antioxidants (zinc, adiponectin, TRAP and SH groups) combined with increased sTNFR2: 98.7% sensitivity, 91.7% specificity, AUC-ROC 0.990. SVM analysis (validation): 93.51% training accuracy, 92.03% validation accuracy. NNA training: sensitivity 98.2%, specificity83.3%, AUC-ROC 0.997	Lower concentrations of all four antioxidants (zinc, adiponectin, TRAP and SH groups) were predictive of MS when compared to controls. TRAP and adiponectin were the most important predictors, followed by zinc and sTNFR2
Goyal et al., Front Neurol. 2019 [ref (40)]	MS: n=910 Healthy volunteers/controls: n=199		Serum cytokines: IL-1β, IL-2, IL-4, IL-8, IL-10, IL-13, IFN-γ, and TNF-α	SVM, DT, RF and neural networks	IL-1β, IL-2, IL-4, IL-8, IL-10, IL-13, IFN-γ, and TNF-α, age, sex, disease duration, EDSS and MSSS (cytokines for MS vs non-MS, and cytokines and other variables for relapsing vs non-relapsing MS)	MS vs non-MS Relapsing vs non-relapsing MS	MS vs non-MS: RF model: sensitivity 75.6%, 85.7% specificity, 90.91% accuracy, ROC-AUC 0.957 Relapsing vs non-relapsing MS: the RF model had the highest accuracy (70%). In the validation set, the RF model was the best discriminator	Cytokines play an important role in the differentiation of Th cells and recruitment of auto-reactive T and B cells in MS
Seitz et al., Ther Adv Neurol Disord. 2021 [ref (42)]	Early MS: n=156: n=110 with no history of ON n=46 with prior history of ON	0	sNfL levels	SVM	sNfL age, sex, disease duration, EDSS	OCT: OPL volume and atrophy	SVM: sNfL levels 75.7% accurate at predicting OPL volume (training 75.9%, testing 76.2%). Longitudinal analysis of sNfL and OPL in ON eyes: sNfL levels 72.1% accurate at predicting OPL atrophy (training 72.5%, testing 71.8%)	NfL was predominantly expressed in the RNFL, GCIPL and OPL in comparison to other layers (murine retina). The findings suggest NfL and OPL associations may be due mostly to inflammation leading to axonal damage
Kosa et al., Nat Commun. 2022 [ref (43)]	MS: n=227 Healthy subjects: n=24		1305 proteins in CSF	RF	Proteins in CSF, age, sex	MS severity: CombiWISE-based MS-DSS at baseline and follow-up, and BVD severity outcome	Training: baseline MS-DSS: 75 unique biomarkers explaining 62% variance. MS-DSS on follow-up, 34 unique biomarkers and 35 for BVD explaining 60% variance. Validation: CSF-based MS-DSS at baseline predicted 17% variance, 26% of MS-DSS at follow-up, 22% of BVD severity model	Identification of 7 patient clusters differing in CSF concentration of proteins from four protein modules (1. Myeloid lineage/TNF; 2. CNS repair; 3. Complement/coagulation; and 4. Adaptive immunity and CNS stress). Cluster 2: predominance of males with progressive MS, relatively low expression in the CNS repair module and high expression in the myeloid lineage/TNF and complement/coagulation modules. These patients had a higher MS severity. Clusters 3 and 4 relatively enriched for female subjects. Cluster 3: high expression of adaptive immunity and CNS module proteins and enriched with relapsing MS subjects. Cluster 4: relatively high expression of all protein modules except for complement/coagulation, with a relatively low MS severity
Gross et al., Brain. 2021 [ref (44)]	Autoimmune neuroinflammatory diseases: n=282 (relapsing MS n=196, NMOSD n=15, Susac syndrome n=14, AE n=57) Degenerative diseases: n=93 (amyotrophic lateral sclerosis n=52, mild Alzheimer´s Disease n=41) Vascular conditions: n=97 Non-inflammatory controls: n=74 (with somatoform disorders or who donated CSF during the course of spinal anesthesia). Total n=546	Additional subjects: n=231 (neuroinflammatory diseases: n=32; neurodegenerative diseases: n=156; neurovascular diseases: n=8; non-inflammatory controls: n=35)	CSF analysis with multiparameter flow cytometry to identify 34 CSF and blood biomarkers after assessing for collinearity	Feature selection with dimensionality reduction and unsupervised cluster analyses	34 CSF and blood features	Neuroinflammatory processes vs other conditions: cells/ml, monocytes, NK cells, and B cells in CSF and CD56dim NK cells in peripheral blood. MS vs other neuroinflammatory disorders: CSF plasma cells and intrathecal IgG synthesis	Neuroinflammatory diseases vs others: 70% sensitivity, 81% specificity, 76% accuracy,ROC-AUC 85% MS vs other neuroinflammatory disorders: Accuracy vs: NMOSD: 87.3%; Susac Syndrome: 95.3%; A E: 89.4%. ROC-AUC vs: NMOSD: 91.5; Susac Syndrome: 90.7; AE: 82.7	MS vs other autoimmune diseases: besides parameters such as intrathecal plasma cells concomitant with IgG synthesis, the analyses identified intrathecal IgA and IgM synthesis. There were other disease-specific parameters, such as alterations in circulating peripheral blood CD56bright NKcells and intrathecal lactate concentrations in NMOSD; circulating CD4+ and CD8+ T cells in Susac Syndrome; and circulating and intrathecal lymphocytes, intrathecal NK T cells, monocytes, and CD14+CD16+ monocytes in AE.

ADAboost-FT, adaptive boosting applied to functional trees; AE, autoimmune encephalitis; AOPP, advanced oxidation protein products; BVD, brain volume deficit; CD, cluster of differentiation; CIS, clinically isolated syndrome; CNN, convolutional neural network; CombiWISE, combinatorial weight-adjusted disability score; CSF, cerebrospinal fluid; DT, decision tree; EDSS, Expandid Disability Status Scale; ESOM, emergent self-organising feature maps; FT, functional trees; GaussianNB, Gaussian Naïve Bayes; GCIPL, macular ganglion cell-inner plexiform layer; GEO, gene expression omnibus data repository; CNS, central nervous system; GTPase, guanosine triphosphate enzyme; HMDD, Human microRNA Disease Database; IFN, interferon; IL, interleukin; miRNA, microRNA; MS, multiple sclerosis; MS-DSS, Multiple Sclerosis Disease Severity Score; MSSS, Multiple Sclerosis Severity Score; NK, natural killer; NMOSD, neuromyelitis optica spectrum disorders; NNA, neural network analysis; OCT, optical coherence tomography; ON, optic neuritis; OND, other neurological diseases; OPL, outer plexiform layer; PBMCs, peripheral blood mononuclear cells; PPMS, primary progressive multiple sclerosis; RBF/SVM, support vector machine with radial basis function; RF, random forests; RNFL, retinal nerve fiber layer; ROC-AUC, receiver-operating characteristic curve-area under the curve; RRMS, relapsing remitting multiple sclerosis; SH, sulphydryl; sNfL, neurofilament light chain in serum; SPMS, secondary progressive multiple sclerosis; sTNFR, soluble tumour necrosis factor receptor; SVM, support vector machine; Th, T helper cells; TNF, tumour necrosis factor; TRAP, total radical-trapping antioxidant parameter.

PBMCs can bear specific dysregulation in genes at different stages of MS. Acquaviva et al. (34) analysed transcriptomic profiles of PBMCs from individuals with CIS (n=57), RRMS (n=108), SPMS (n=26), PPMS (n=35), OND (n=27), and HC (n=60), divided into training (n=224) and validation (n=89) datasets. They defined classifiers (MS vs non-MS, relapsing vs progressive MS) using nested cross-validation in the training dataset. Then they used ward DT-based algorithms [RF, functional trees (FTs) and adaptive boosting applied to FT (ADAboost-FT) to evaluate their performance in the validation dataset. ADAboost-FT generated the best model to differentiate MS from non-MS (94.3% sensitivity, 87.5% precision). Identified transcripts in MS were related to interferon signalling, chromatin remodelling, and apoptosis. The relapsing vs progressive MS classifier showed 83.3% sensitivity and 93.8% precision. Associated biological themes included cell cycle and T cell activation for both progressive forms; protein ubiquitination, cell migration, and fatty acid metabolism for PPMS; and GTPase activity regulation, locomotor behaviour, and blood coagulation in SPMS.

MicroRNAs (miRNAs) play critical roles in post-transcriptomal gene expression regulation. In MS, miRNAs have been implicated in various aspects of the disease’s pathophysiology (35). Sun et al. (36) proposed a convolutional neural network (CNN)-based model to identify MS-related miRNAs and compared it to other existing methods: DT, SVM, logistic regression, and Gaussian Naïve Bayes. Using the miRNA-MS associations from the Human microRNA Disease Database (HMDD), the CNN model showed the highest ROC-AUC (0.87). Some of the top 10 predicted miRNAs were differentially expressed in RRMS or related to Th17 cell differentiation, whereas another one decreased after initiation of therapy with interferon β.

Lipid metabolism may influence inflammation, neurodegeneration, myelin damage, and repair processes in MS (37). Lötsch et al. (38) used unsupervised ML implemented as emergent self-organising feature maps (ESOM) combined with the U*-matrix visualisation technique to analyse eicosanoids, ceramides, and lysophosphatidic acids in serum of 102 PwMS and 301 HC, to find distance and density-based structures. Clear data structures were observed in eicosanoid and ceramide concentrations. Whereas the classification of MS vs HC yielded a moderate performance with eicosanoids (54% sensitivity, 100% specificity, 77% accuracy) the structures emerging with ceramides resulted in a high performance (89.2% sensitivity, 100% specificity, 94.6% accuracy).

An imbalance of oxidant and antioxidant molecules has been implicated in demyelination and axonal damage in MS. Mezzaroba et al. (39) used supervised ML (neural network analysis [NNA] and SVM with radial basis function [RBF/SVM]) to evaluate discriminatory patterns in plasma of 9 oxidants and antioxidants and zinc serum levels, in 174 PwMS and 182 controls. The combination of low levels of four antioxidants and increased levels of one oxidant yielded the best prediction for MS (sensitivity 98.7%, specificity 91.7%, AUC-ROC 0.990). The SVM analyses obtained 93.51% training and 92.03% validation accuracies *(* 39 *).*

Cytokines play an important role in Th cell differentiation and recruitment of auto-reactive T and B cells in MS. Goyal et al. (40) used four ML models (SVM, DT, RF, and neural networks) to identify serum cytokines predictive of MS. They also assessed the cytokines with age, sex, disease duration, EDSS, and MSSS to classify MS into remitting and non-remitting MS. They used 910 serum samples from PwMS and 199 from HC (total n=1109). Of these, 900 were included in the training set and 209 in the testing set. RF was the model that best predicted MS (sensitivity 75.6%, specificity 85.7%, accuracy 90.91%, ROC-AUC 0.957) and also had the highest accuracy (70%) to differentiate relapsing from non-relapsing MS. In the validation set, the RF model was again the best discriminator (40).

Neurofilament light chain (NfL) is a biomarker of axonal damage in MS (41). Seitz et al. (42) used SVM analysis to test for associations between baseline serum NfL (sNfL) and different retinal thickness measures in 156 early MS patients: 110 with no history of optic neuritis (ON) and 46 with ON. After adjusting for age, sex, disease duration, and EDSS, a significant correlation was found only between high sNfL levels and low outer plexiform layer (OPL) volume in patients with a history of ON. Follow-up OCTs available for 38 subjects with a mean (SD) follow-up of 2.1 (1.4) years showed baseline sNfL correlated with absolute OPL atrophy in ON. sNfL levels predicted OPL volume with 75.9% training and 76.2% testing accuracies. In the longitudinal analysis, sNfL predicted OPL atrophy with 72.5% training and 71.8% testing accuracies.

Other studies have focused on CSF biomarkers. Kosa et al. (43) used RF to search for biomarkers among 1305 proteins in CSF of 227 PwMS to build models predictive of disease severity. To differentiate natural aging and sex effects from MS-related mechanisms they used data from 24 HC. MS severity was assessed using the combinatorial weight-adjusted disability score (CombiWISE)-based MS Disease Severity Score (MS-DSS) measured at baseline and follow-up, and the brain volume deficit (BVD) severity outcome, based on linear regression models of brain parenchymal fraction and age, calculated from MRIs performed within 3 months of CSF collection. Initial analyses demonstrated positive associations of coagulation and complement cascades and negative associations for NOTCH signalling and neuron recognition categories with MS severity. After adjusting for age and sex, the model selected 75 biomarkers explaining 62% of variance for baseline MS-DSS. For follow-up MS-DSS, 34 biomarkers were selected and 35 for BVD explaining 60% of variance. The effect sizes decreased to 17%, 26%, and 22% of variance in the validation cohort (n=98). Using unsupervised cluster analyses, the authors identified seven patient clusters differing in CSF protein concentrations from four protein modules. Of note, one cluster had a predominance of men with progressive MS, a relatively low expression in the CNS repair module and high expression in the myeloid lineage/TNF and complement/coagulation modules. These patients had a higher MS severity.

Cellular characterisation in blood and CSF can help differentiate between CNS disorders and clarify their pathophysiological processes. Gross et al. (44) combined feature selection with dimensionality reduction and unsupervised cluster analyses to investigate parameters altered across autoimmune neuroinflammatory diseases [RRMS n=196, neuromyelitis optica spectrum disorders (NMOSD) n=15, Susac syndrome n=14, autoimmune encephalitis (AE) n=57], other CNS conditions (neurodegenerative n=93, vascular n=97), and non-inflammatory controls (n=74) (total n=546). The validation cohort included 231 additional subjects (neuroinflammatory n=32, neurodegenerative n=156, neurovascular n=8, non-inflammatory controls n=35). Exploratory analyses identified four CSF parameters and one peripheral blood parameter that together discriminated neuroinflammatory diseases from other groups (70% sensitivity, 81% specificity, 76% accuracy, ROC-AUC of 85%). When aiming to differentiate MS from other neuroinflammatory diseases, CSF plasma cells and intrathecal IgG synthesis alone were sufficient to distinguish RRMS from other neuroinflammatory diseases with high accuracy and ROC-AUC (NMOSD: 87.3% and 91.5%; Susac syndrome: 95.3% and 90.7%; AE: 89.4% and 82.7%). Finally, the authors compared cell profiles in RIS, CIS and early RRMS (≤36 months from disease onset) vs late RRMS (>36 months). Alterations in the proportions of CD56dim NK cells and biomarkers of intrathecal inflammation gradually increased during disease evolution. When splitting RRMS based on inflammatory activity, minor effects were shown in most intrathecal parameters, whereas changes in peripheral and intrathecal CD4+CD8+ T cells and intrathecal plasma cells were more pronounced.

## Limitations of AI-based research in MS fluid biomarkers

AI-based studies using fluid biomarkers in MS offer promising results. However, these studies have limitations which are worth being mentioned. In general, all these studies still have relatively small sample sizes, which, together with the lack of external validation analyses in many of them, limit the generalisability of the results. Also, despite the low number of studies published so far, there is a large methodological variability, which, at times, is not explained in detail, making it very difficult to replicate the analyses done (**Tables 1** **–** **3**). These limitations are common to all AI-based studies that harness biomarker data to improve the diagnosis, predict or understand the disease, thus hampering the application of all these models to clinical practice.

In relation to the specific limitations of those studies focused on diagnosis, the number and types of diseases which have been compared with are limited. Furthermore, many of the tests (biomarkers) used by the authors are not available in routine clinical practice. These aspects reduce the utility of these models in practice, at least in the short term, suggesting the need for more research.

Regarding the studies focused on prediction of disease evolution, apart from the general limitations abovementioned, many of them have cross-sectional designs or, if they have a longitudinal design, there is a relatively short follow-up time in most of the cases. Also, very often, the effect of treatment is not taken into account. Furthermore, most studies were not adjusted for important demographic, clinical and technical aspects, such as race, ethnicity, disease duration, brain volume, and the interval between sampling and relapses or their treatment. Finally, despite the developments in AI-based models in MS which use raw neuroimaging and deep learning techniques to predict clinical outcome, the integration of these into AI-based models which use fluid biomarkers (or the other way around) is still lacking. Little is known about the complementary roles of both types of predictors and the potential synergies between them. However, it is highly likely that only when both are used together in comprehensive models, a real impact on the clinical management of MS can be achieved. Such integration requires, though, intensive methodological research which will hopefully bear fruit in the near future.

Lastly, regarding the limitations of the studies focused on understanding disease mechanisms, many of them are far too focused on certain paths or predictors, therefore not allowing us to explain or understand the whole picture. Also, very importantly, the fact that many of these biomarkers, paths, or predictors, may explain the same variance of a given outcome measure but we are not aware of that – because typically one study tends to focus on a given path – implies that many of the associations found may be reflecting mere epiphenomena rather than causally related events. Whereas this might be less relevant for building predictive models, for those studies which aim at understanding the disease through AI, this may be deleterious.

## Conclusions and future directions

The application of AI-based methodologies to tackle key challenges in MS is exponentially increasing. However, in this context, the number of studies published in the literature focusing on the use of fluid biomarker data is still small. Most of these publications are focused on serum biomarkers, genetic variants, and gene expression profiles as predictors. Of note, only half of them have included an external validation analysis of the developed AI model, thus hampering a full interpretation of the results and their potential generalisability.

Importantly, after the assessment of the papers published so far, it may be said that the research on AI applied to biomarker data is still quite in its early days and that we are still far from clinical applications. So far, AI methodologies have been very useful for biomarker discovery in MS, but the large heterogeneity of methods and results suggests that we may need many years of research before prototypes can be launched to help healthcare professionals and patients in the clinic.

Along the same lines, even though many studies reported much higher accuracy levels when fluid biomarker, MRI, and clinical data were combined as predictors of diagnosis or disease evolution, large studies combining the most important types of predictor acquired in the clinic are lacking. Only when these take place and are replicated in large independent cohorts will we be able to comprehend their full potential and start considering that a change in patient management thanks to the introduction of those AI-based models is possible. Of note, for these models to be useful in the clinic, they need to use, as input data (predictors), routinely-acquired biomarkers, including laboratory, imaging, and clinical data. On the other hand, it is possible that a branch of AI-based research in MS, i.e., that focused on understanding the pathogenic mechanisms and those processes underlying disability accumulation, continues to exist with the use of less common (non-routinely acquired) biomarkers. This research is also important and will surely bring to light crucial knowledge on the disease, essential for its ultimate eradication. A final conclusion is that all studies carried out so far confirm the leading role of inflammatory pathways in MS.

Future directions include the development of larger studies with validation in independent datasets. Also, future directions should aim at the design of longitudinal studies with longer follow-ups (for those mainly focused on future prediction), hopefully accounting for the complex effects of disease-modifying treatments and other dynamic data, as well as the integration of fluid biomarkers, neuroimaging, optical coherence tomography (OCT) imaging, and clinical predictor data to build robust and powerful models.

Furthermore, forthcoming research endeavours must transition from the current exploratory phase of AI-based methodologies applied to biomarker data in MS to a more translational stage. This shift necessitates thorough evaluation of the clinical utility of the constructed AI models. For that, the future lies in creating guidelines for AI-based analyses to improve the comparability across studies, to shed light on the steps needed to go from discovery to clinical practice implementation, and to evaluate utility of AI-based algorithms in practice. Additionally, we should be able to learn from AI-based investigations on other neurodegenerative diseases (45) to overcome the challenges surrounding these types of studies.

As a final consideration, it is imperative to recognise that addressing ethical and inequality concerns surrounding AI-based analyses is just as crucial as resolving technical challenges. With the exponential growth of AI studies, maintaining research integrity in AI research demands not only initial attention but also ongoing evolution, keeping pace with the rapid advancement of science to meet the needs and expectations of us all.
